# Adjuvant chemotherapy omission after pancreatic cancer resection: a French nationwide study

**DOI:** 10.1186/s12957-024-03393-7

**Published:** 2024-05-06

**Authors:** Charles Poiraud, Xavier Lenne, Amélie Bruandet, Didier Theis, Nicolas Bertrand, Anthony Turpin, Stephanie Truant, Mehdi El Amrani

**Affiliations:** 1 Digestive Surgery and Transplantation Department, CHU de Lille, 59000 Lille, France; 2 University of Lille, 59000 Lille, France; 3 Department of Medical Information, CHRU de Lille, 59000 Lille, France; 4 University of Lille, 59000 Lille, France; 5 Medical Oncology Department, CHU de Lille, 59000 Lille, France; 6 University of Lille, 59000 Lille, France; 7 Service de Chirurgie Digestive Et Transplantation, Hôpital CLAUDE HURIEZ, Rue Michel Polonovski LILLE CEDEX, 59037 Lille, France

**Keywords:** Pancreatic adenocarcinoma, Adjuvant chemotherapy, Pancreatic cancer surgery, Hospital volume

## Abstract

**Background:**

Adjuvant chemotherapy (AC) improves the prognosis after pancreatic ductal adenocarcinoma (PDAC) resection. However, previous studies have shown that a large proportion of patients do not receive or complete AC. This national study examined the risk factors for the omission or interruption of AC.

**Methods:**

Data of all patients who underwent pancreatic surgery for PDAC in France between January 2012 and December 2017 were extracted from the French National Administrative Database. We considered “omission of adjuvant chemotherapy” (OAC) all patients who failed to receive any course of gemcitabine within 12 postoperative weeks and “interruption of AC” (IAC) was defined as less than 18 courses of AC.

**Results:**

A total of 11 599 patients were included in this study. Pancreaticoduodenectomy was the most common procedure (76.3%), and 31% of the patients experienced major postoperative complications. OACs and IACs affected 42% and 68% of the patients, respectively. Ultimately, only 18.6% of the cohort completed AC. Patients who underwent surgery in a high-volume centers were less affected by postoperative complications, with no impact on the likelihood of receiving AC. Multivariate analysis showed that age ≥ 80 years, Charlson comorbidity index (CCI) ≥ 4, and major complications were associated with OAC (OR = 2.19; CI_95%_[1.79–2.68]; OR = 1.75; CI_95%_[1.41–2.18] and OR = 2.37; CI_95%_[2.15–2.62] respectively). Moreover, age ≥ 80 years and CCI 2–3 or ≥ 4 were also independent risk factors for IAC (OR = 1.54, CI_95%_[1.1–2.15]; OR = 1.43, CI_95%_[1.21–1.68]; OR = 1.47, CI_95%_[1.02–2.12], respectively).

**Conclusion:**

Sequence surgery followed by chemotherapy is associated with a high dropout rate, especially in octogenarian and comorbid patients.

**Supplementary Information:**

The online version contains supplementary material available at 10.1186/s12957-024-03393-7.

## Introduction

Despite tremendous efforts to improve prognosis, pancreatic ductal adenocarcinoma (PDAC) is expected to be the second leading cause of cancer [1]. After curative resection, adjuvant chemotherapy (AC) can significantly improve disease-free and overall survivals [2]. Since the CONKO-001 trial, gemcitabine for 6 months was the recommended regimen after resection until the FOLFIRINOX regimen became the current standard [3].

AC is considered an important component of multimodal PDAC treatment. However, previous studies have shown that most patients undergoing curative-intent resection do not receive AC after surgery. Indeed, a recent study by Bertens et al*.* reported that nearly half of patients failed to receive AC after surgery for PDAC [4]. The main reasons given were related to the occurrence of postoperative complications and poor general condition. Furthermore, early interruption of adjuvant chemotherapy (IAC) has a significant negative impact on the survival of PDAC patients [5].

Previous studies have identified predictive factors for not receiving AC after pancreatectomy for PDAC [6, 7]. Most of these studies focused on factors related to patients without considering the impact of hospital characteristics. However, we previously reported that postoperative outcomes after pancreatectomy were considerably correlated with hospital volume [8]. This volume-outcome relationship after pancreatectomy can be cautiously extrapolated to AC. However, the correlation between hospital volume and AC remains unknown.

Therefore, this nationwide study investigated the factors associated with the likelihood of receiving and completeness of AC. We also aimed to study the variation between the hospital volume and AC after pancreatectomy for PDAC. The results could help to identify reasons for underutilization of AC to improve the outcomes of patients with pancreatic cancer.

## Patients and methods

### PMSI database

Data were extracted from the French National Administrative Database for Hospital Care (Programme de Médicalisation des Systèmes d’Information, PMSI) as described elsewhere [9]. This database includes summaries of all hospital stays in France and links each admission to the same patient. All diagnoses and therapeutic procedures were carefully collected and summarized using a dedicated coding system. Discharge abstracts included patient demographics, diagnosis (based on the International Classification of Diseases, 10th edition [ICD-10]), and therapeutic procedures (based on the Classification of Commune des Actes Médicaux [CCAM]) [10]. The relevance and reliability of this dataset for clinical studies have been validated multiple times [11].

### Study population

Patients who underwent pancreatoduodenectomy (PD), distal pancreatectomy (DP), or total pancreatectomy (TP) for pathologically confirmed PDAC with curative intent between January 2012 and December 2017 were included. We used ICD-10 to define all primary diagnoses identified during admission for elective pancreatectomy. The exclusion criteria were exploratory laparotomies, bypass procedures, and death within 90 postoperative days (POD), age below 18 years, residence outside metropolitan France, and an incorrect patient identifier. The study complied with French National Health guidelines on research involving human subjects. IRB approval was not needed for this study.

### Study variables

Data regarding demographic characteristics (age, sex, nutritional status, and obesity), surgical and postoperative courses (type of procedure, need for vascular resection, intensive care unit (ICU) stay, postoperative complications), and hospital characteristics (type of facility and hospital volume) were extracted from the PMSI database. Comorbidities were weighted using the validated Charlson comorbidity index (CCI), and patients were further stratified into four groups according to surgical risk (0,1,2–3, > 4) [12]. Other variables analyzed were sex, age, neoadjuvant chemotherapy, malnutrition according to French guidelines [13], obesity and surgical procedure.

We categorized postoperative complications as minor or major complications up until 90 days after pancreatectomy. As previously described, we considered a major complication if the patient required readmission to the step down care unit (SDCU) with at least one complication encoded or if the patients required critical care in the ICU. We also considered a major complication if it required a reoperation procedure. Reoperation was defined as a surgical, endoscopic, or radiological procedure performed postoperatively. Minor complications included any complications that did not lead to ICU admission or a second stay in the SDCU [14]. According to our previous study, we used the discriminant threshold of 26 annual pancreatectomies to define a low (> 26 cases/year) and high (≥ 26 cases/year) volume center [8].

During the study period, the recommended protocol in the adjuvant setting was gemcitabine, started within 3 months and continued for 6 months (18 courses). Thus, we considered as “OAC” all patients who failed to receive at least one course of gemcitabine within 12 postoperative weeks. IAC was defined as less than 18 courses of AC, regardless of the duration of treatment. Any chemotherapeutic treatment received by the patient before surgery was considered neoadjuvant chemotherapy.

### Statistical analysis

Categorial variables were compared using the Khi-square test of Pearson and given as a percentage. Quantitative variables were compared using Fisher’s exact test and expressed as means and standard deviations. We performed multivariate analysis using the logistic regression method and included the following variables: surgical volume, age, comorbidity, type of pancreatectomy, vascular resections, and neoadjuvant chemotherapy. The tests were bilateral, and the level of statistical significance was set at 5%. Stata version 13.1 (StataCorp, College Station, TX, USA) was used for all analyses.

## Results

### Description of the study population

A total of 11 599 patients underwent pancreatic surgery for PDAC in France, between January 2012 and December 2017. The main characteristics of the patients are summarized in Table 1. The mean age of the patients was 66 years, and 53.5% were men. Twelve percent (*n* = 1401) received neoadjuvant chemotherapy before surgery. PD was the most common procedure (76.3%), and 19.5% of patients required vascular resection. Sixty percent of the patients experienced at least one postoperative complication, and half of these complications were considered as major (Table 1).

**Table 1:**
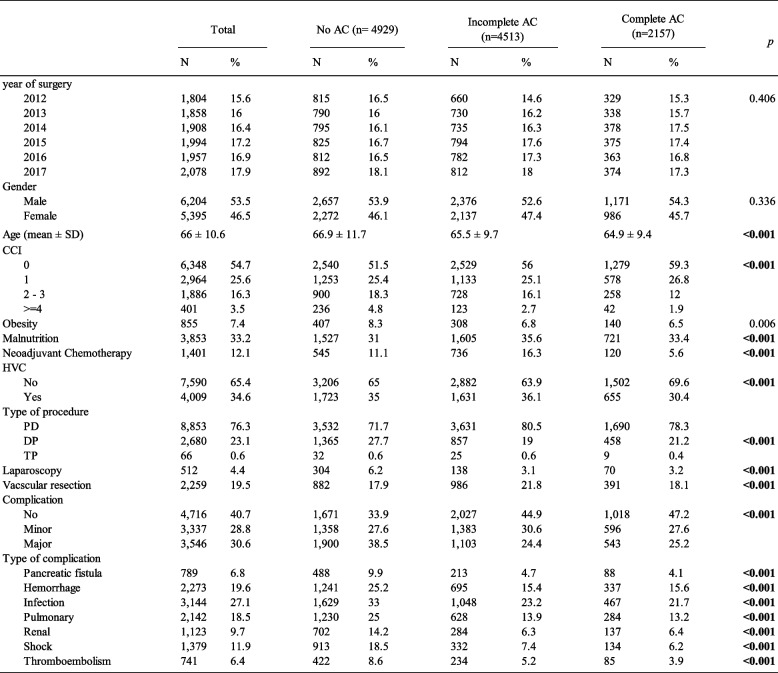
Characteristics of the 11,599 patients operated on for PDAC between 2012 and 2017 in France, stratified according to the receipt of adjuvant chemotherapy

*AC* Adjuvant chemotherapy, *SD* Standard deviation, *CCI* Charlson comorbidity index, *HVC* High Volume Center, *PD* Pancreaticoduodenectomy, *DP* Distal Pancreatectomy, *TP* Total pancreatectomy

Overall, 4929 patients (42.5%) did not receive any course of chemotherapy within 12 postoperative weeks; therefore, the AC exposure rate was 57.5%. Moreover, approximately two-thirds of the patients (67.7%) were affected by IAC. Ultimately, only 18.6% of the cohort completed adjuvant treatment. The absolute number of pancreatectomies for cancer increased over the study period (1804 in 2012 and 2078 in 2017), but the rates of OAC and IAC remained stable (*p* = 0.406).

### Surgical volume

Thirty-four percent of patients underwent surgery in a high-volume center (Table 2). No differences in age or comorbidities were found between the high-and low-volume centers. However, malnutrition was more common in patients who underwent pancreatectomy at high-volume centers (40% vs. 29%, *p* < 0.001). Moreover, neoadjuvant treatment and vascular resection were performed more frequently in high-volume centers than in low-volume centers (17.5% vs. 9.2%, *p* < 0.001 and 26% vs. 16%, *p* < 0.001, respectively).Finally, the rate of postoperative complications was lower in high-volume centers (56.2% vs. 61%, *p* < 0.001), especially major complications (26.6% vs. 32.6%, *p* < 0.001).

**Table 2:**
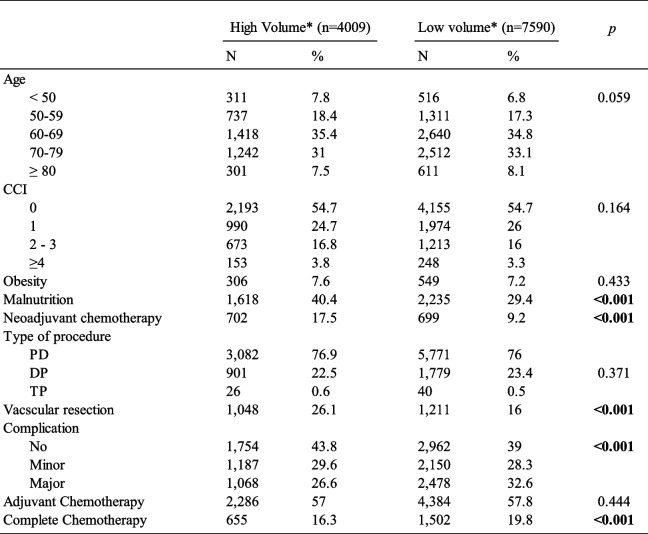
Surgical volume of centers

*High Volume Centers were defined as those who perform more than 26 pancreatic resection annually (see Method); *CCI* Charlson comorbidity index, *PD* Pancreaticoduodenectomy, *DP* Distal Pancreatectomy, *TP* Total pancreatectomy

### Risk factors for OAC

The risk factors associated with OAC are presented in Table 3. Univariate analysis revealed that age ≥ 80 years ( odds ratio (OR) = 2.27; CI_95%_ [1.87–2.76]), CCI ≥ 4 (OR = 2.14; CI_95%_ [1.75–2.63]), and the occurrence of a major postoperative complication (OR = 2.1; CI_95%_ [1.92–2.30]) were the main risk factors for OAC.

**Table 3:**
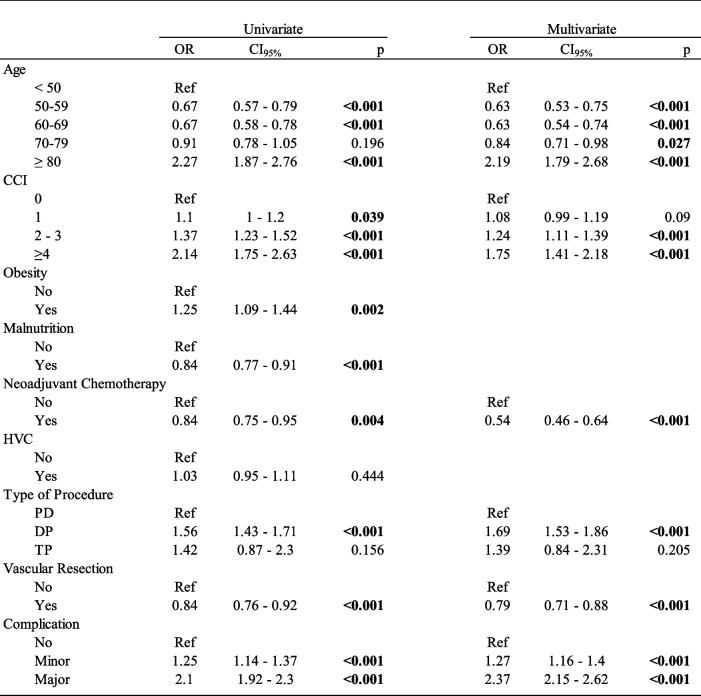
Uni-and multivariate analysis of risk factors for omission of adjuvant chemotherapy

*OR* Odds Ratios, *CI*_95%_ 95% Confidence Interval, *CCI* Charlson comorbidity index, *HVC* High Volume Center, *PD* Pancreaticoduodenectomy, *DP* Distal Pancreatectomy, *TP* Total pancreatectomy

Neoadjuvant chemotherapy was associated with lower OAC (OR = 0.84; CI_95%_ [0.75–0.95]) along with pancreatectomy with vascular resection (OR = 0.84; CI_95%_ [0, 76–092]). Interestingly, surgical volume had no impact on the likelihood of receiving AC.

On multivariate analysis, most of these results were confirmed: age ≥ 80 years, CCI ≥ 4, and major complications were independently associated with OAC (OR = 2.19; CI_95%_ [1.79–2,68]; OR = 1.75; CI_95%_ [1.41–2.18] and OR = 2.37; CI_95%_ [2.15–2.62], respectively), while neoadjuvant chemotherapy and vascular resection remained associated with a lower risk of OAC. Surprisingly, we found that DP was an independent risk factor for OAC (OR = 1.69; CI_95%_ [1.53–1.86]).

### Risk factors for IAC

Logistic regression analysis showed that age ≥ 80 years (OR = 1.54; CI_95%_ [1.1 – 2.15]), CCI 2–3 (OR = 1.43; CI_95%_ [1.21–1.68]), and CCI ≥ 4 (OR = 1.47; CI_95%_ [1.02–2.12]) were also independent risk factors for IAC (Table 4). Similarly, neoadjuvant chemotherapy, high hospital volume, and vascular resection were associated with an increased likelihood of IAC (OR = 1.78; CI_95%_ [1.42–2.24], OR = 1.2; CI_95%_ [1.01–1.44] and OR = 1.19; CI_95%_ [1.03–1.37], respectively). Postoperative complications, either major or minor, were not associated with ICA (OR = 0.99; CI_95%_ [0.86–1.13], OR = 1.12; CI_95%_ [0.99–1.27] respectively).

**Table 4:**
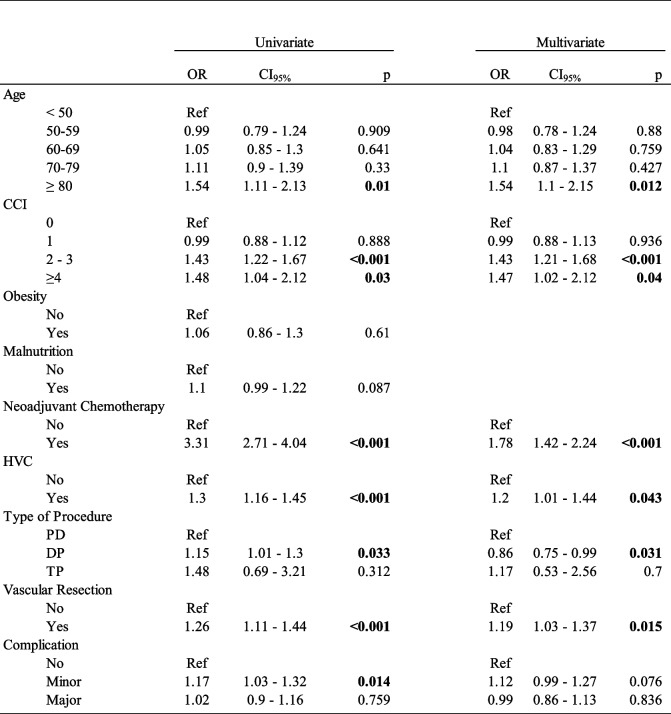
Table 4 Uni- and multivariate analysis of risk factors for interruption of adjuvant chemotherapy

*OR* Odds ratios, *CI95%* 95% Confidence Interval, *CCI* Charlson comorbidity index, *HVC* High Volume Center, *PD* pancreaticoduodenectomy, *DP* Distal Pancreatectomy, *TP* Total pancreatectomy

## Discussion

In the current study, we used an administrative database to identify factors associated with adjuvant chemotherapy after pancreatectomy for PDAC. We showed that approximately 40% of the patients undergoing pancreatectomy for PDAC failed to receive AC. Additionally, in two-thirds of the cases, treatment was prematurely stopped. Elderly patients and those with comorbidities were less likely to have AC. Similarly, age ≥ 80 years and CCI ≥ 2 were risk factors for IAC. In view of these worrying findings, management of patients with PDAC should be considered.

The results of the current study are consistent with those of other population-based studies conducted in Europe and the United States. Altmanet al. reported that in the MEDICARE population, 35% of patients received AC, and only 7% completed the full course [15]. However, other countries have reported better AC rates after pancreatic cancer resection. In a recent study from the Dutch Pancreatic Cancer Audit, the rate of OAC use was 33% [7]. Similarly, a multicenter study in Japan showed that 66% of patients received AC [16].

Our results showed that for octogenarians, the risk of OAC was double, and 70% of those who initiated it did not complete the treatment. Previous studies have reported similar results, suggesting an effect of age on patient selection for AC [17, 18]. Several reasons could be advocated to explain this finding. First, the occurrence of postoperative morbidity increases in older patients, leading to a longer postoperative recovery. Second, poor tolerance to severe adverse effects and altered quality of life are more likely to occur in older patients, which contributes to limited access to AC for these patients. Third, the elderly are largely underestimated in clinical trials, in which the findings are not necessarily applicable to these patients. In France, it is recommended that all patients deemed fit should receive six months of AC after surgery regardless of age and tumor stage [19]. During the study period, 18 courses of gemcitabine were recommended, primarily based on the CONKO-001 trial [20]. This trial only included patients who had fully recovered from surgery, in good general condition with good bone marrow function and no active infection, impaired coagulation or renal function, etc. Although these conditions are requisite for a trial, the majority of the patients were excluded. These findings are worrying, given the worse prognosis and poor survival benefit of surgery alone compared to palliative chemotherapy in the octogenarian population (13 vs. 10 months, respectively, in the retrospective analysis by Marmor et al*.* [18].

In the current study, approximately one-third of patients experienced major complications after pancreatectomy. Interestingly, we found that the risk of OAC was 2.34 fold higher after major complications. This correlation was previously reported by Labori et al. [21]. The authors reported that oth initiation and completion rates of AC were significantly lower in patients with postoperative major complication. Importantly, postoperative complications are also associated with a higher rate of recurrence and worse prognosis after PDAC resection [6, 22]. In addition to increasing the risk of OAC, postoperative complications can induce immune suppression by inflammation and associated immunological phenomena leading to worse oncological outcomes [23, 24]^.^

Interestingly, we have shown that vascular resection was associated with a higher rate of IAC but tended to decrease the risk of OAC. This trend has already been observed in a large American retrospective study by DePeralta et al*.* [25]*.* Perhaps patients selected for these difficult surgeries had a better baseline general condition and were more frequently treated with neoadjuvant chemotherapy. In addition, these findings may be driven by the disease burden and toxicity of extended chemotherapy. Thus, the performance of vascular resections did not limit the ability to initiate AC but was associated with an increased risk of chemotoxicity.

Distal pancreatectomies are less morbid than PD, and we believe that the risk of OAC would be lower. Our data refute this hypothesis and show a significantly higher risk of OAC use after DP. This trend seems surprising, but has already been reported. Bergquist et al. found a similar OAC rate between DP and PD in a retrospective analysis of 13 501 pancreatectomies (33.7% and 32%, respectively; *p* = 0.148) [26]. Note that in this study, the readmission rate did not differ between the DP and PD groups (8.7% vs. 8%, respectively; *p* = 0.386). Thus, DP is perhaps responsible for more occult and less severe adverse events than DP but may postpone the onset of AC. Diabetes, for instance, is significantly more common after DP than after PD [27] and can interfere with the initiation of AC. We also found that thromboembolic complications were more likely to occur after DP than after PD (8.2% vs. 5.9%, *p* < 0.001, Supplemental Table 1). Furthermore, thromboembolic complications were independently associated with OAC use (odds ratio [OR],1.86; 95%[1.6—2.17], Supplemental Table 2).

The results of our study should be interpreted considering several limitations. The retrospective and administrative approaches are exposed to selection bias and coding errors. However, the PMSI database is the basis of the financial allocation for all health establishments. Consequently, the French Ministry of Health frequently checks the quality of encoding. In addition, we only focused on this study on patients who received gemcitabine chemotherapy in order to ensure greater uniformity. However, FOLFIRINOX regimen is considered as the current standard for adjuvant chemotherapy after pancreatectomy for PDAC. Therefore, the results of this study should be carefully interpreted. However, in France, all cancer cases are submitted to a multidisciplinary board. Therefore, the proportion of patients treated outside the recommendations (clinical trials) should be marginal. Finally, PMSI is an administrative database that lack detailed clinical information regarding oncological characteristics of tumors and survival data of patients. Despite these limitations, the current study is the first to report the rates of omission and interruption of adjuvant chemotherapy after pancreatectomy for cancer in France.

## Conclusion

We showed that omission and interruption of adjuvant treatment were common after pancreatectomy for pancreatic cancer in France. Nearly 80% of the patients who underwent surgery failed to complete the postoperative chemotherapy protocol. Therefore, new strategies are necessary to improve chemotherapy exposure in potentially curable patients.

### Supplementary Information

**Supplementary Material 1.**

## Data Availability

No datasets were generated or analysed during the current study.
