# Supplementary material for: Adjuvant chemotherapy omission after pancreatic cancer resection: a French nationwide study
Source: World J Surg Oncol. 2024 May 6;22:123. doi: 10.1186/s12957-024-03393-7 (PMC11071280; doi:10.1186/s12957-024-03393-7)
Supplement: Supplementary file 1 — Supplementary Material 1. [file 12957_2024_3393_MOESM1_ESM.docx]

# Supplemental Tables
